# Enhancing Functional Properties and Protein Structure of Almond Protein Isolate Using High-Power Ultrasound Treatment

**DOI:** 10.3390/molecules29153590

**Published:** 2024-07-30

**Authors:** Li Tian, Xinyong You, Shulin Zhang, Zhenbao Zhu, Jianhua Yi, Gang Jin

**Affiliations:** 1 College of Biology and Food Engineering, Anyang Institute of Technology, Anyang 455000, China; litian@ayit.edu.cn (L.T.); xinyong8206@163.com (X.Y.); shulinzhng@gmail.com (S.Z.); 2 College of Horticulture and Landscape Architecture, Henan Institute of Science and Technology, Xinxiang 453003, China; 3 School of Food and Biological Engineering, Shaanxi University of Science and Technology, Xi’an 710021, China; zhuzhenbao@sust.edu.cn; 4 Guangxi Key Laboratory of Quality and Safety Control for Subtropical Fruits, Guangxi Subtropical Crops Research Institute, Nanning 530001, China

**Keywords:** high-power ultrasound, almond protein isolate, physicochemical protein properties, protein functional properties

## Abstract

The suitability of a given protein for use in food products depends heavily on characteristics such as foaming capacity, emulsifiability, and solubility, all of which are affected by the protein structure. Notably, protein structure, and thus characteristics related to food applications, can be altered by treatment with high-power ultrasound (HUS). Almonds are a promising source of high-quality vegetable protein for food products, but their physicochemical and functional properties remain largely unexplored, limiting their current applications in foods. Here, we tested the use of HUS on almond protein isolate (API) to determine the effects of this treatment on API functional properties. Aqueous almond protein suspensions were sonicated at varying power levels (200, 400, or 600 W) for two durations (15 or 30 min). The molecular structure, protein microstructure, solubility, and emulsifying and foaming properties of the resulting samples were then measured. The results showed that HUS treatment did not break API covalent bonds, but there were notable changes in the secondary protein structure composition, with the treated proteins showing a decrease in α-helices and β-turns, and an increase in random coil structures as the result of protein unfolding. HUS treatment also increased the number of surface free sulfhydryl groups and decreased the intrinsic fluorescence intensity, indicating that the treatment also led to alterations in the tertiary protein structures. The particle size in aqueous suspensions was decreased in treated samples, indicating that HUS caused the dissociation of API aggregates. Finally, treated samples showed increased water solubility, emulsifying activity, emulsifying stability, foaming capacity, and foaming stability. This study demonstrated that HUS altered key physicochemical characteristics of API, improving critical functional properties including solubility and foaming and emulsifying capacities. This study also validated HUS as a safe and environmentally responsible tool for enhancing desirable functional characteristics of almond proteins, promoting their use in the food industry as a high-quality plant-based protein.

## 1. Introduction

Ultrasound treatment is an innovative technology that is often employed to change or measure the characteristics of food products and is therefore widely applied in the food industry [1]. Generally, ultrasound treatments can be categorized as one of two types: low-frequency high-power ultrasound (classified as frequencies from 20 to 100 kHz with a power from 10 to 1000 W cm^−2^) and high-frequency low-power ultrasound (classified as frequencies > 100 kHz with power intensities < 1 W cm^−2^) [2]. In the food industry, high-powered ultrasound (HUS) is mainly utilized to alter food properties, whereas low-powered ultrasound is employed to measure food qualities [3,4,5]. HUS can be employed in various food applications including crystallization, sterilization, fermentation, homogenization, extraction, and de-aggregation [6]. In these contexts, HUS functions by forming micro-streaming currents and causing cavitation in liquids [7]; bubbles formed via cavitation collapse suddenly, causing extremely high pressure and temperatures at those sites (about 5000 K and about 1000 atm, respectively) [8,9]. These factors cause alterations in the physical qualities of processed foods [10]. In addition, ultrasonic waves can facilitate the generation of highly reactive free radicals from water molecules (H_2_O → •H+•OH); these unstable free radicals can chemically modify surrounding molecules [11,12].

As a result of its capacity to cause such chemical and physical modifications, HUS has long been used to improve or alter properties such as gelling, foaming, interaction capacities, and solubility in animal and plant proteins. For example, whey protein isolates and concentrate treated with HUS have significantly enhanced solubility and foaming properties compared to untreated samples [1]. Wheat gluten proteins can also be improved by HUS; it is presumed that ultrasonic waves change their molecular structure [13]. Through similar changes, ultrasonic treatment can enhance the solubility and hydrophobicity of black bean proteins [14]. Peanut proteins can form complexes with dextran, which promotes the solubility and emulsification of the proteins; the rate of this process can be increased by HUS treatment [15]. Globular proteins isolated from soy also show HUS-induced increases in gelling and water retention capacity [16]. Although numerous such studies have demonstrated improvement in food protein properties as a result of ultrasonic treatment, few studies have addressed the effects of HUS on almond proteins.

Almonds are extremely popular throughout the world for their pleasant flavor, high nutritional quality, and health benefits [17]. Due to this popularity, they are the most abundantly produced tree nut worldwide; 1.06 million tons were produced globally in 2016. Almonds have high protein content (~16–22%) with proteins that contain all essential amino acids. Although the fat content is very high (~50–55%), most of these fats (90%) are unsaturated, and almonds are cholesterol-free. These characteristics make them an appealing choice as a source of plant-based proteins.

Almond kernels (whole, powdered, cut, chopped, or ground) are commonly used in baking [18], wherein they provide a texture that many consumers find pleasant. This texture arises partially from the specific functional properties of almond proteins [19]. One major protein makes up ~65% of the total protein content, with the remainder comprising several small, water-soluble minor proteins [18,20]. Then, almond protein isolate (API) has been widely used in functional and nutritional food ingredients due to its affordability and excellent nutritional content. However, API is less able to form foam, and the foam it does form is less stable [17], which leads to poor functional characteristics of the protein. Recently, ultrasound technology has been extensively applied to enhance the functional properties of plant protein isolates, such as whey [1], *Prunus persica* kernel [11], black bean [14], soy protein isolates [16], and so on, but few studies in the literature have focused on the characteristics of API using HUS treatment. Here, we investigated the effects of HUS on the molecular and physicochemical properties of API. We proposed that, similar to other popular food proteins, the functional properties of API could be improved by HUS treatment, thereby enabling almonds to be utilized in a variety of new applications within the food industry.

## 2. Results and Discussion

### 2.1. Analysis of Processed Almond Flour

An analysis of the purified defatted almond flour was conducted following AOAC standards [21]. The sample was demonstrated to possess 0.64% ash, 89.56% protein, and 0.85% fat. This indicated the high purity of the protein sample and the successful elimination of the fat content, indicating that these samples were appropriate for the subsequent analyses.

### 2.2. Impacts of HUS on API Structure

#### 2.2.1. Molecular Weight (MW)

**Figure 1:**
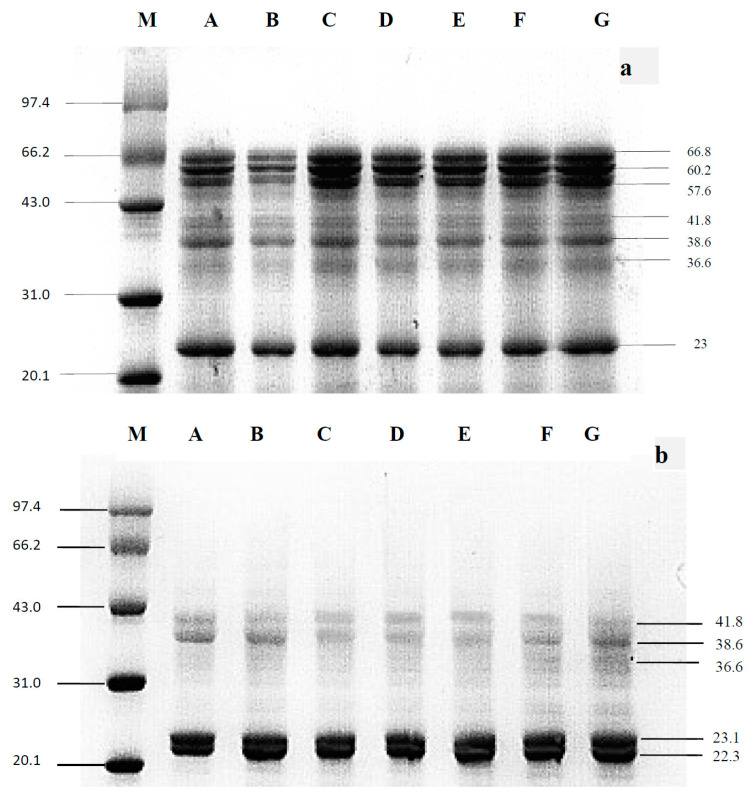
Standard and reducing sodium dodecyl sulfate–polyacrylamide gel electrophoresis (SDS-PAGE) of almond protein isolate (API). (**a**) Standard and (**b**) reducing gel conditions. Lane M, marker; Lane A, untreated control sample; Lanes B–G, API samples treated with high-power ultrasound (HUS) at 200 W for 15 min (Lane B), 200 W for 30 min (Lane C), 400 W for 15 min (Lane D), 400 W for 30 min (Lane E), 600 W for 15 min (Lane F), and 600 W for 30 min (Lane G).

To determine the impact of HUS on almond protein properties, the MW profiles of treated and untreated proteins were first assessed with both standard and reducing SDS-PAGE (Figure 1). Under standard (non-reducing) conditions (Figure 1a), both sample types showed seven high-power bands each, corresponding to 23.0, 36.6, 38.6, 41.8, 57.6, 60.2, and 66.8 kDa. These results were consistent with almond proteins identified previously [18,22], with no significant changes in MW between the HUS-treated and the untreated samples, suggesting that sonication did not cleave peptide bonds in any of the isolated proteins. Previous studies of proteins from other organisms, such as rice and soybean, have also found that sonication does not alter MW profiles [23]. However, in contrast, HUS treatment could decrease the MW of α-albumin and proteins extracted from jackfruit seeds in other research studies [24,25]. These inconsistencies between studies indicate that HUS-induced protein fragmentation likely depends on variables including the type of protein, the solution in which the protein is suspended, and the sonication parameters. Notably, the SDS-PAGE bands in the HUS-treated samples were more intense than those in the untreated control, which may be due to the increased water solubility of the treated proteins.

Just like the results obtained under standard SDS-PAGE conditions [22,23], the protein bands produced by HUS-treated samples showed no significant change compared with untreated samples under reducing SDS-PAGE (Figure 1b). This suggested that peptide or disulfide bonds in the almond protein isolated were not disrupted by HUS treatment. Although there were no significant differences between treated and untreated samples under either set of SDS-PAGE conditions, there were differences between samples run on the standard gel compared to those run on the reducing gel. Specifically, under reducing conditions, the bands at 57.6, 60.2, and 66.8 kDa showed increased power, whereas the band at 22.3 kDa was less intense compared to the standard SDS-PAGE. This may have been due to the reducing gel containing β-mercaptoethanol, which breaks the disulfide bonds that commonly hold together high-MW polypeptide complexes [18]. Overall, these results indicated that HUS treatment had no significant impact on the MW of almond proteins, likely because the peptide bonds were not cleaved due to the formation of large polypeptide complexes through disulfide bonds.

#### 2.2.2. Secondary Structure

The secondary structures of HUS-treated and untreated almond proteins were next analyzed with far-UV CD spectroscopy. In contrast to the insignificant results of sonication on peptide bonds, HUS markedly altered the protein secondary structures (Table 1). Specifically, increases in the power and duration of sonication were associated with decreased α-helices and β-turns, as well as increased random coil content, suggesting that HUS treatment promoted protein unfolding. Because protein secondary structures are determined by hydrogen bonding [26], these results indicated that HUS strongly disrupted specific types of hydrogen bonds in the almond proteins, particularly those involved in forming α-helices and β-turns. Similarly, Jiang et al. [14] reported that the HUS treatment of black bean proteins also reduced the proportion of α-helices, although they also reported an increased proportion of β-sheets. Sun et al. [27] found that HUS decreased the content of α-helices and β-sheets, while the β-turns and random coil content increased. Other previous studies have shown that lower-power sonication led to reductions in α-helix and random coil content in soy protein, but higher-power sonication resulted in increases in those same structures, such as in the case of HUS treatment where the content of α-helices and β-turns decreased [28]. These varied results further indicate that the impact of sonication on protein properties depends on several factors, such as the type of protein, the suspension solution, and sonication parameters. However, the secondary structure results of the present study offered the first evidence of significant alterations in almond proteins as a result of HUS treatment.

#### 2.2.3. Surface Free SH Groups

**Figure 2:**
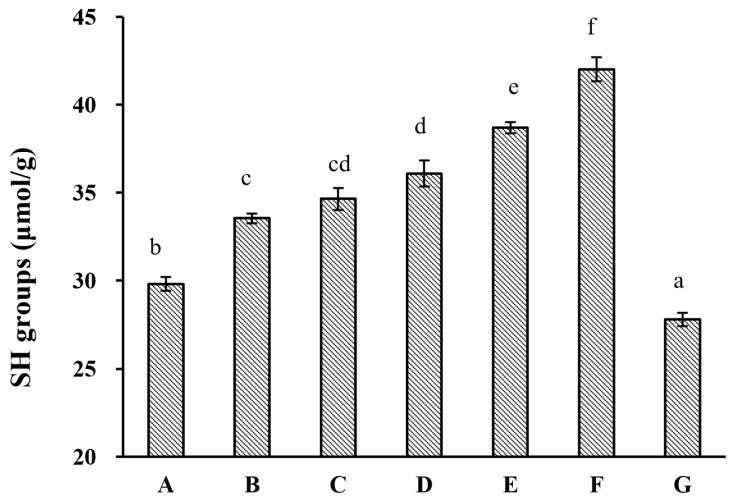
Surface sulfhydryl (SH) group content in almond protein isolate (API) samples. (**A**) Free surface SH content in untreated control samples. (**B**–**G**) Free surface SH content in API samples treated with high-intensity ultrasound (HUS) at (**B**) 200 W for 15 min, (**C**) 200 W for 30 min, (**D**) 400 W for 15 min, (**E**) 400 W for 30 min, (**F**) 600 W for 15 min, and (**G**) 600 W for 30 min. Lowercase letters above each bar represent statistical significance groups at *p* < 0.05.

HUS-induced structural changes in almond proteins were further investigated by measuring free SH group content on the protein surfaces (Figure 2). This analysis showed that HUS caused significant elevations in the surface free SH content, except in the samples subjected to treatment at 600 W for 30 min. This was largely consistent with the previous reports of sonication treatment increasing free SH levels in samples of walnut and sesame proteins [29,30]. These increases could have occurred because S–S bonds were broken by sonication, which allowed for the subsequent formation of new SH groups. However, the lack of alterations to the protein MW profiles as a result of HUS treatment (Figure 1) made this explanation unlikely. Alternatively, HUS-induced partial protein unfolding could have exposed previously buried free SH groups [30]. Other researchers have also reported decreases in free SH levels as a result of sonication treatment [26], consistent with the drastic decrease observed here in free SH content in samples treated at 600 W for 30 min. Long HUS treatments can generate reactive H_2_O_2_, which may react with free SH groups [30,31]; this could cause decreases such as those that we found here in the sample treated with the highest intensity for the longest time. Furthermore, according to the reduction in free sulfhydryl groups, another common index of protein oxidation, the API surface free SH groups increasing under HUS treatment indicated that HUS treatment could improve the antioxidant properties of almond protein isolate (API), which was advantageous for preserving the quality and stability of protein emulsions when incorporating API into food products.

#### 2.2.4. Intrinsic API Fluorescence

**Figure 3:**
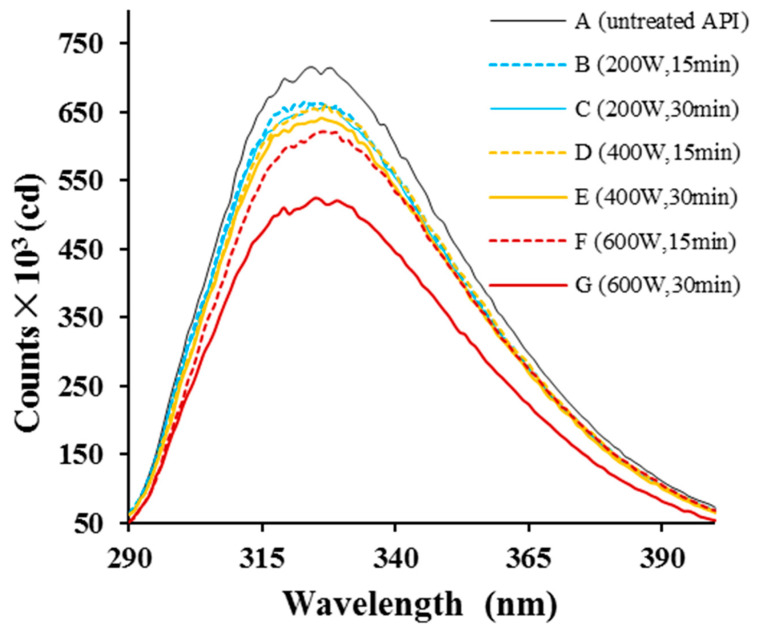
Intrinsic fluorescence emissions from almond protein isolate (API) samples. (**A**) Fluorescence spectrum from untreated control samples. (**B**–**G**) Fluorescence spectra from API samples treated with high-intensity ultrasound (HUS) at (**B**) 200 W for 15 min, (**C**) 200 W for 30 min, (**D**) 400 W for 15 min, (**E**) 400 W for 30 min, (**F**) 600 W for 15 min, and (**G**) 600 W for 30 min.

We next assessed changes in API structure by measuring the intrinsic protein fluorescence (Figure 3). Structural changes that affect aromatic residues (Tyr, Phe, and Trp) can alter the fluorescence spectrum of a protein. Here, both HUS-treated and control API samples showed peak fluorescence emission at ~326 nm. However, the maximum peak height was lower in samples treated with higher intensities or for longer durations, demonstrating that the treatment did indeed alter API structure, aggregation, or both [32], changing the local environment surrounding Tyr, Phe, and/or Trp residues. Similarly, egg and soy proteins have been shown to have lower fluorescence intensity after sonication treatment [33,34]. Overall, the analyses conducted to this point suggested that sonication altered API secondary and tertiary structures.

### 2.3. Impacts of HUS on API Microstructure

#### 2.3.1. Protein Suspension Particle Size

Particle size can strongly influence protein functional properties [35]. We therefore measured the mean diameters of API samples (D_43_ and D_32_) before and after undergoing HUS treatment (Table 2). Notably, it was significantly reduced with HUS treatment (Table 2). In the most striking alternation, D_43_ and D_32_ decreased from ~89.77 μm and ~10.60 μm, respectively, before HUS treatment to ~20.01 μm and ~4.97 μm after treatment at 400 W for 30 min, respectively. The decreases in particle size were likely caused by aggregate disruption as a result of cavitation and other HUS-induced forces [36]. The results were consistent with prior research on whey protein and soybean glycinin [37]. However, we found that HUS at high intensity was less efficient for aggregate disruption; the mean particle sizes of API samples treated at 600 W were larger than those treated with lower intensities. This may have been because strongly denatured proteins tended to aggregate. This hypothesis is supported by Zhang et al. [32], who found that sonicating oil-in-water emulsions at very high intensities increased the particle size, and another study in egg white protein showed increased particle sizes after HUS [37]. These results indicate that optimal sonication parameters for protein aggregate disruption are likely to vary based on the type of protein, the protein conformation, and the degrees of denaturation and aggregation.

#### 2.3.2. Lyophilized API Microstructure

**Figure 4:**
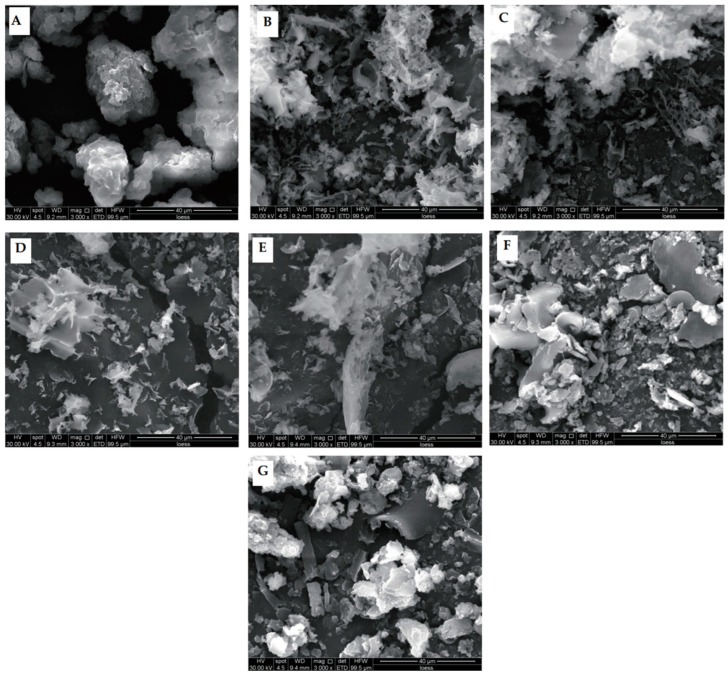
Microstructure of lyophilized almond protein isolate (API) samples as visualized with scanning electron microscopy. (**A**) Structure of untreated control API sample. (**B**–**G**) Structures of representative API samples treated with high-intensity ultrasound (HUS) at (**B**) 200 W for 15 min, (**C**) 200 W for 30 min, (**D**) 400 W for 15 min, (**E**) 400 W for 30 min, (**F**) 600 W for 15 min, and (**G**) 600 W for 30 min.

Powdered API samples were visualized with SEM to determine the effects of sonication on protein microstructure (Figure 4). Untreated samples appeared lumpy, and the edges clearly showed multiple layers. However, HUS treatments dramatically changed the protein structures, yielding more irregular shapes. For example, HUS treatments at lower intensities (200–400 W) caused sample breakage and a fluffier appearance. These results were consistent with those discussed above demonstrating increased particle breakage resulting from HUS treatments with a high intensity or a long duration. However, the highest-intensity treatment (600 W) caused one sample to form a sheet and parts of another sample to appear clumpy, similar to the untreated samples; this suggested large aggregate formation. Similar findings of low-intensity HUS decreasing fragment size but higher-intensity treatments causing larger fragment sizes have been reported for chicken myofibrillar protein [25]. In summary, SEM demonstrated that HUS strongly affected lyophilized API structure, which in turn may have affected protein functionality.

### 2.4. Impacts of HUS on API Functional Properties

#### 2.4.1. Solubility

**Figure 5:**
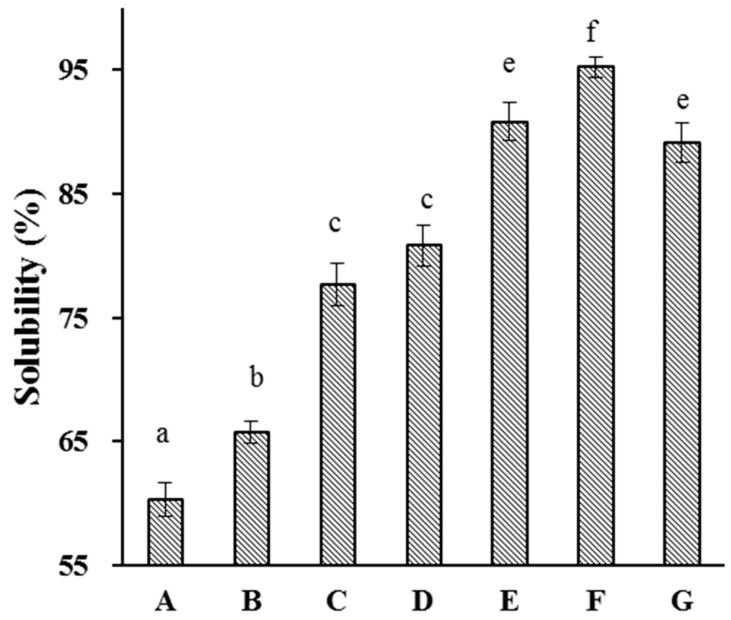
Solubility of almond protein isolate (API) samples. (**A**) Solubility of untreated control API samples. (**B**–**G**) Solubility of API samples treated with high-intensity ultrasound (HUS) at (**B**) 200 W for 15 min, (**C**) 200 W for 30 min, (**D**) 400 W for 15 min, (**E**) 400 W for 30 min, (**F**) 600 W for 15 min, and (**G**) 600 W for 30 min. Lowercase letters above each bar represent statistical significance groups at *p* < 0.05.

Proteins used in food applications are preferred to be highly water-soluble; this property allows them to be readily used in applications such as gelling and emulsifying [38]. We therefore investigated the effects of HUS on API water solubility (Figure 5). API water solubility tended to significantly increase along with HUS power and treatment time. For example, the control sample had the lowest water solubility (60.4%), whereas the sample treated at 600 W for 15 min had the highest water solubility (95.2%). This was consistent with previous reports of increases in the water solubility of whey and myofibrillar proteins after sonication treatment [39,40,41]. The enhancement of API water solubility may be attributed to the disruption of physical bonds between protein molecules by HUS, reducing aggregate formation and promoting increased interactions between the proteins and water molecules [37]. It is also possible that HUS caused structural changes affecting the chemistry of surface residues, altering their water solubility [42].

#### 2.4.2. Emulsifiability

**Figure 6:**
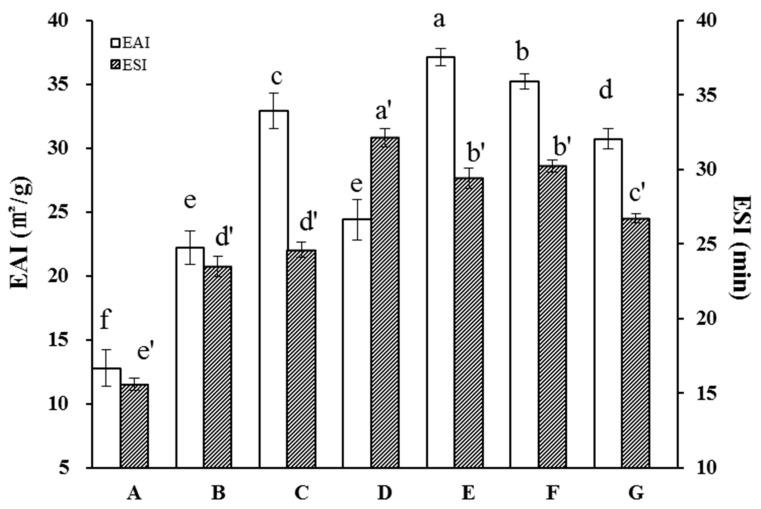
Emulsifying activity index (EAI) and emulsifying stability index (ESI) values of almond protein isolate (API) samples. (**A**) EAI and ESI values for untreated control API samples. (**B**–**G**) EAI and ESI values for API samples treated with high-intensity ultrasound (HUS) at (**B**) 200 W for 15 min, (**C**) 200 W for 30 min, (**D**) 400 W for 15 min, (**E**) 400 W for 30 min, (**F**) 600 W for 15 min, and (**G**) 600 W for 30 min. Lowercase letters above each bar represent statistical significance groups at *p* < 0.05.

Emulsifiability is a critical characteristic of the proteins used in food products of essentially all types [35]. Previous studies have demonstrated improvement in protein emulsification characteristics as a result of sonication, including in sunflower, soy, and whey proteins [43,44,45]. We therefore measured the effects of HUS on API emulsification properties (Figure 6). Overall, the EAI was notably elevated in HUS-treated samples relative to the control, demonstrating that HUS treatment enhanced their capacity to form emulsions. HUS power and treatment time were associated with an increased EAI; the latter increased from about 12.8 m^2^/g in the control to a peak of 37.4 m^2^/g in the sample treated at 400 W for 30 min. The increases in the EAI among treated API samples may have resulted from altered surface chemistry or the increased proportion of small soluble proteins that adsorbed to the surface between the water and oil.

In addition to the EAI, we measured the ESI in the API samples. As with the EAI, this parameter was uniformly higher among the treated samples than in the control samples. For example, the ESI increased from ~12.8 ± 1.4 min in the control sample to a peak of 37.1 ± 0.7 min in the sample treated with 400 W for 30 min. This may have occurred because the emulsions formed by HUS-treated samples formed smaller, more stable droplets; alternately, differences in lipid droplet chemistry may have changed the attractive forces between droplets [46,47]. Notably, the highest HUS intensity was associated with a decrease in the ESI compared to the 400 W treatment. As discussed above, this may have been a result of the high levels of protein unfolding and subsequent aggregation.

Protein emulsifiability can be influenced by the hydrophobic properties of the surface [48]. Typically, optimized surface hydrophobicity is necessary for a protein to have both high water solubility and favorable surface activity. As discussed above, HUS can cause partial protein unfolding, particularly in globular proteins, which can expose hydrophobic groups that are usually buried in the protein interior, thus increasing surface activity [49]. Whether alterations in surface hydrophobicity were responsible for the increases in API emulsifiability should be addressed in a future study.

#### 2.4.3. API Foaming Properties

**Figure 7:**
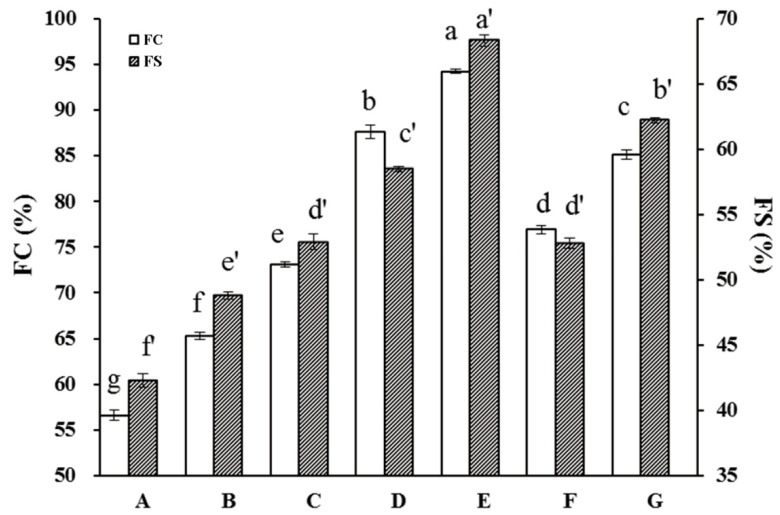
Foaming capacity (FC) and foaming stability (FS) characters of almond protein isolate (API) samples. (**A**) FC and FS values for untreated control API samples. (**B**–**G**) FC and FS values for API samples treated with high-intensity ultrasound (HUS) at (**B**) 200 W for 15 min, (**C**) 200 W for 30 min, (**D**) 400 W for 15 min, (**E**) 400 W for 30 min, (**F**) 600 W for 15 min, and (**G**) 600 W for 30 min. Lowercase letters above each bar represent statistical significance groups at *p* < 0.05. Error bars show standard deviation.

Stable foam formation is another important functional protein property for food applications. Here, the FC was found to be significantly improved by HUS treatment (Figure 7); untreated samples averaged FC values of 56.6 ± 0.6%, compared to the maximum FC of 94.2 ± 0.7% in the sample treated with 400 W for 30 min. HUS also significantly enhanced the FS of all treated samples, with the highest values observed at 400 W for 30 min. These results suggested that HUS positively influenced both the FC and FS of API. Similar effects have also been demonstrated in pea proteins, gourd seed protein, and jack bean seed protein isolate [50,51,52].

Protein interfacial adsorption is important for foam formation and stabilization, but this characteristic is highly dependent on protein size, flexibility, and surface hydrophobic properties [53]. Sonication, here, reduced the particle size of API, which led to an increased air–water interface, thus improving the foaming properties [32]. In addition, we observed that HUS treatment caused partially unfolded protein structures (Table 1), which likely exposed an increased number of hydrophobic regions [54]. Theoretically, this would increase the protein capacities for adsorbance at the air–water interface, promoting foaming [1]. Overall, consistent with other properties such as foaming stability and emulsifiability, they were here found to be increased in APIs via HUS treatment, although the underlying mechanisms require further study.

## 3. Material and Methods

### 3.1. Reagents

Ellman’s reagent was purchased from Sigma Aldrich (St. Louis, MO, USA). Sodium dodecyl sulfate, β-mercaptoethanol, bovine serum albumin (BSA), ethylenediaminetetraacetic acid (EDTA), glycine, and Coomassie brilliant blue G250 were obtained from the MedChemExpress (Shanghai, China). Other reagents were purchased from Moshake Biotechnology Co. Ltd. (Wuhan, China). Analytical-grade chemicals were used for all experiments.

### 3.2. API Preparation

Defatted almond flour was generated based on the previously described methods [18,55], with some modifications. Briefly, almond kernels were purchased from local suppliers. The kernels were peeled and stirred constantly in petroleum ether with a ratio of 1:6 almonds/solvent (*w/v*) for 24 h. This formed a slurry, which was vacuum-filtered; the filtered almond material was then subjected to a second extraction in petroleum ether and vacuum-filtered. Subsequently, the material was dried at 50 °C, ground into flour, and passed through a size 40 mesh. The flour was collected and stored at −20 °C prior to further experiments.

API was prepared using a modified approach based on the method described by Sze-Tao and Sathe [17]. The defatted almond flour was mixed with deionized water at a 1:15 ratio (*w/v*) and adjusted to a pH of 8.0 with 0.5 M NaOH. The almond–water slurry was heated to 45 °C and stirred for one hour, and then centrifuged at 8000× *g* and 4 °C for 10 min. After the supernatant was removed, the pellet was extracted in water twice more following the same steps. After the third extraction in water, the mixture was adjusted to a pH of 4.0 with 0.5 M HCl and stirred for one hour at room temperature (RT), and then centrifuged at 8000× *g* and 4 °C for 10 min. The resulting pellet was blended with deionized water and agitated for one hour. The pH was adjusted to 7.0 with 0.5 M NaOH, and then the sample was dialyzed for 48 h at 25 °C with dialysis bags (molecular mass cutoff 8 kDa, Biosharp Co., Hefei, China). The purified API sample was then lyophilized and stored at −20 °C for further analysis.

### 3.3. HUS Treatment

API dispersions (0.5% *w/v*) were generated in 10 mM phosphate buffer (pH 7.0) to a final volume of 200 mL with stirring at RT for one hour. The resulting samples were processed at 25 kHz with an XH-300UA ultrasound machine (Biosharp Co., Hefei, China) containing a titanium probe 1.8 cm in diameter. Samples in 250 mL quartz glass flasks were sonicated with 200, 400, or 600 W for 15 or 30 min based on the method reported by Zhu et al. [30]. The pulse durations were two-second ON with one-second OFF intervals between pulses. The treated samples were immediately used in the measurements described below.

### 3.4. API Structural Measurements

#### 3.4.1. SDS–Polyacrylamide Gel Electrophoresis (SDS-PAGE)

The molecular weight (MW) values of sonicated and control API samples were analyzed with both standard and reducing SDS-PAGE as previously described [56]. Briefly, API samples from each treatment group were prepared by adding 400 μL of the lyophilized protein at 2.5 mg/mL to 400 μL of buffer and 400 μL of deionized water. The protein mixtures were boiled at 95 °C for 5 min, and then 10 μL per sample was loaded into a 5% stacking and 15% separating gel. Electrophoresis was conducted at a constant voltage of 160 V. Smart View software version FR-980A (Furi Science & Technology Co., Ltd., Shanghai, China) was used to capture and analyze the gel image.

#### 3.4.2. Circular Dichroism (CD) Analysis

Changes in the secondary protein structures induced by sonication were analyzed using CD. Each API sample was suspended in a 10 mM phosphate buffer with pH 7.0 to a final concentration of 0.1 mg/mL before being filtered through a 0.22 µm filter. Phosphate buffer alone was used as the blank control for subtraction from the sample spectra. Samples were placed in cells with path lengths of 0.1 cm immediately after filtration. CD spectra were obtained at 190–260 nm using a Jasco J-810 spectropolarimeter (Tokyo, Japan) at RT with the following parameters: step resolution = 0.5 nm; scan rate = 100 nm/min; bandwidth = 1.0 nm; and response time = 0.25 s. For each sample, spectra were obtained in technical octuplicate and averaged into a single final spectrum. The software provided by the spectropolarimeter manufacturer (Tokyo, Japan) was used to analyze the spectra and identify secondary structures, namely random coils, α-helices, β-sheets, and β-turns.

#### 3.4.3. Fluorescence Emission Spectroscopy

To analyze intrinsic API fluorescence, the samples were suspended in a phosphate buffer (pH 7.0) to 1.5 mg/mL, and then centrifuged with 10,000× *g* at RT for 10 min. The supernatant was removed and excited at 295 nm (with a 3.0 nm bandwidth) in an FS-5 fluorescence spectrophotometer (Edinburgh, UK). Emission spectra were collected at 300–400 nm (with a 2.0 nm bandwidth).

#### 3.4.4. Surface Free Sulfhydryl (SH) Group Analysis

The SH group content on almond protein surfaces was measured as previously described by Zhu et al. [30] with some modifications. Briefly, API samples were suspended in 0.15% (*w/v*) Tris-glycine buffer. Ellman’s reagent (50 μL) was mixed with 5 mL of each API suspension, and then the samples were incubated with shaking in a water bath at 25 ± 1 °C for one hour. After incubation, the samples were centrifuged at 10,000× *g* at RT for 10 min, and A412 values were collected from the supernatant using a UV–visible spectrophotometer (Genesys, ThermoSpectronic, Waltham, MA, USA). A buffer with Ellman’s solution only was measured as a blank. Free SH content was calculated in μmol/g of protein using a molar extinction coefficient of 13,600 M/cm.

### 3.5. API Microstructure Analysis

#### 3.5.1. Particle Size Measurements

The mean particle diameter and particle size distribution were measured in HUS-treated and untreated API samples with a Mastersizer 2000 particle sizing instrument (Malvern Instruments Ltd., Malvern, UK). Samples were added into a stirred measurement cell containing 700 mL deionized water until the light obscuration reached ~10–15%. Particle sizes in each sample were calculated and expressed as the volume-weighted mean diameter (D_43_) and as the surface-weighted mean diameter (D_32_).

#### 3.5.2. Scanning Electron Microscopy (SEM)

SEM was used to visualize the overall structure of lyophilized treated and untreated API. Samples were evenly distributed on a conductive adhesive, and then an ion sputter coater was used to coat the samples with gold under argon. Sample images were taken at 25 kV under 3000× magnification (ZEISS, Jena, Germany).

### 3.6. Functional Property Measurements

#### 3.6.1. Solubility Analysis

Protein solubility was measured as described by Hu et al. [57] with some modifications. HUS-treated and untreated API samples were centrifuged at 8000× *g* at RT for 10 min. Coomassie brilliant blue was used to generate a BSA standard curve, from which API concentrations were calculated in each suspension before centrifugation and in the supernatant after centrifugation. Protein solubility was calculated as the proportion of protein in the supernatant out of the total protein content. There were three biological replicates per treatment group.

#### 3.6.2. Emulsification Properties

The emulsifying activity index (EAI) and emulsifying stability index (ESI) were calculated for HUS-treated and untreated API samples as previously described [58] with some modifications. API (0.5% *w/v*) dispersions (40 mL per sample) were combined with 10 mL soybean oil. The mixtures were homogenized at 35,000 rpm for 2 min in an F6/10-G Ultra-Turrax homogenizer (Ika, Germany). From the resulting emulsion, 50 μL per sample was immediately removed from the bottom of each container and mixed with 10 mL of 0.1% SDS. Absorbance at 500 nm was immediately measured (A0), and then measurements were taken again after 10 min (A10). These values were used to calculate the EAI and ESI as follows:

(1) $$\mathrm{EAI}\;(m^{2}/g)=\frac{2\;\times \;2.303\;\times \;\mathrm{A0}\;\times \;\mathrm{DF}}{C\;\times \;\Phi \;\times \;\theta \;\times \;10,000}$$

(2) $$\mathrm{ESI}\;\left(\min\right)=\frac{\mathrm{A0}}{\mathrm{A0}-\mathrm{A10}}\;\times \;10$$

in which DF means the dilution factor (200); C means the protein concentration (g/mL); θ means the light path length (1 cm); and Φ means the oil volume fraction (0.2).

#### 3.6.3. Foaming Capacity (FC) and Foaming Stability (FS)

The FC and FS were measured as described by Duan et al. [54] with slight modifications. Sonicated and control API solutions (50 mL each at 0.5% *w/v*) were homogenized with an M133/1281-0 hand-held mixer (Biospec Products, Inc., Bartlesville, OK, USA) at 15,000 rpm for 2 min under RT conditions. The resulting foamed samples were placed in 100 mL cylinders and incubated at RT for 30 s prior to measuring the total volume of each sample (in mL). After an additional incubation at RT for 30 min, the FC and FS were calculated as follows:

(3) $$\mathrm{FC}\;(\%)\;=\frac{A-B}{B}\times 100$$

(4) $$\mathrm{FS}\;(\%)\;=\frac{\mathrm{AC}-B}{B}\times 100$$

A is the volume after mixing; B is the volume before mixing; and C is the volume after incubation at RT for 30 min.

### 3.7. Statistical Analyses

Unless otherwise stated, all measurements were taken in technical triplicate. The mean values and standard deviation were calculated from these replicate measurements. The differences between samples were assessed with a one-way analysis of variance (ANOVA) followed by post hoc Duncan’s multiple range test in SPSS v19.0 (SPSS, Inc., Chicago, IL, USA). The differences were considered statistically significant at *p* < 0.05.

## 4. Conclusions

HUS was found to strongly influence API particle size, structure, and functional properties, increasing water solubility, emulsifying properties, and foaming properties partially as a result of the decreased particle size. We suppose that these outcomes resulted from the disruption of within-protein physical bonds, which caused protein denaturation and the formation of smaller complexes. Parameters such as the sonication intensity and treatment duration remain to be optimized, and the specific physical changes underlying functional alterations should be clarified in the future. However, the results of this study could satisfy the comprehensive needs of manufactured protein products and provide the theoretical basis and direction for the API application; further investigation into various properties is necessary to fully understand the detailed mechanism.

## Figures and Tables

**Table 1:** Effects of high-power ultrasound (HUS) treatment on secondary structure composition (%) of almond protein isolate (API) samples.

Sample	α-Helix (*%*)	β-Sheet (*%*)	β-Turn (*%*)	Random Coil (*%*)
Untreated API	35.4 ± 0.3 ^a^	11.5 ± 0.3 ^d^	23.1 ± 0.1 ^a^	30.0 ± 0.3 ^f^
200 W for 15 min	32.6 ± 0.1 ^b^	11.4 ± 0.2 ^d^	22.9 ± 0.3 ^a^	33.1 ± 0.3 ^e^
200 W for 30 min	32.2 ± 0.1 ^c^	11.5 ± 0.2 ^cd^	22.0 ± 0.2 ^b^	34.5 ± 0.3 ^c^
400 W for 15 min	30.9 ± 0.3 ^c^	11.6 ± 0.3 ^bc^	22.1 ± 0.3 ^b^	35.4 ± 0.3 ^d^
400 W for 30 min	29.8 ± 0.3 ^d^	11.9 ± 0.3 ^b^	22.2 ± 0.1 ^b^	36.1 ± 0.3 ^b^
600 W for 15 min	28.0 ± 0.2 ^e^	11.3 ± 0.1 ^a^	22.0 ± 0.2 ^b^	38.7 ± 0.3 ^b^
600 W for 30 min	25.8 ± 0.1 ^f^	11.4 ± 0.3 ^a^	19.4 ± 0.3 ^c^	43.4 ± 0.3 ^a^

Superscript letters within each column indicate statistical significance groups at *p* < 0.05.

**Table 2:** Volume-weighted mean diameter (D_43_) and surface-weighted mean diameter (D_32_) characters of almond protein isolate (API) particles under high-power ultrasound (HUS) treatment.

Sample	D_43_ (μm)	D_32_ (μm)
Untreated API	89.77 ± 0.39 ^a^	10.60 ± 0.12 ^a^
200 W for 15 min	79.29 ± 0.57 ^b^	9.79 ± 0.15 ^b^
200 W for 30 min	62.65 ± 0.51 ^d^	8.10 ± 0.19 ^c^
400 W for 15 min	68.86 ± 0.66 ^c^	6.84 ± 0.32 ^d^
400 W for 30 min	20.01 ± 0.47 ^f^	4.97 ± 0.14 ^g^
600 W for 15 min	47.66 ± 0.53 ^e^	6.02 ± 0.20 ^f^
600 W for 30 min	49.21 ± 1.01 ^e^	6.57 ± 0.11 ^e^

Superscript letters within each column indicate statistical significance groups at *p* < 0.05.

## Data Availability

The original contributions presented in the study are included in the article, further inquiries can be directed to the corresponding authors.
